# La chirurgie laparoscopique de *downstaging* pour cancer colorectal avec métastases hépatiques synchrones: quel intérêt dans les hépatectomies en deux temps? à propos d’une série de 6 cas

**DOI:** 10.11604/pamj.2023.46.38.35775

**Published:** 2023-09-27

**Authors:** Zaki Boudiaf, Chafik Bouzid, Mohamed Rafik Ait-arab, Karim Cherchar, Mohand Kheloufi, Aissam Chibane, Ihsene Hatem Boutekedjiret, Zakia Hattou, Fatiha Gouaref, Kamel Bentabak

**Affiliations:** 1 Service de Chirurgie Oncologique A, Centre Pierre et Marie Curie (CPMC), Avenue Bouzenad Salem, 16000 Alger, Algérie,; 2 Faculté de Médecine d´Alger, Université Benyoucef Benkhedda, Alger, Algérie,; 3 Etablissement Hospitalier Spécialisé en Lutte Contre le Cancer de Blida, Blida, Algérie,; 4 Faculté de Médecine, Université Saad Dahleb Blida I, Blida, Algérie

**Keywords:** Laparoscopie, métastases hépatiques synchrones, ligature portale, Laparoscopy, synchronous liver metastases, portal vein ligation, colorectal cancer, two-stage hepatectomy

## Abstract

Les métastases hépatiques bilobaires des cancers colorectaux posent un problème de prise en charge, la chirurgie curative nécessite souvent plusieurs temps. Le but de notre étude était d´évaluer l´approche laparoscopique avec ligature portale dans le premier temps des hépatectomies en deux temps pour les métastases hépatiques synchrones des cancers colorectaux (MHSCCR). Il s´agissait d´une étude rétrospective monocentrique d´août 2016 à janvier 2020 qui avait inclus les patients présentant des MHSCCR nécessitant une chirurgie curative en deux temps à cause d´une insuffisance du volume du futur foie restant (FFR). L´objectif principal était d´évaluer la morbi-mortalité postopératoire du premier temps laparoscopique à 30 jours. Les objectifs secondaires étaient d´évaluer le taux de conversion, l´hypertrophie du FFR après ligature portale laparoscopique, la morbi-mortalité postopératoire du 2^e^ temps hépatique et enfin d´évaluer le taux d´achèvement de la stratégie thérapeutique. Nous avons inclus six patients (4 hommes et 2 femmes), l´âge moyen était de 64 (44-72) ans, le premier temps opératoire avait consisté en une résection colique laparoscopique associée à une ligature portale droite chez 5 patients et gauche chez un patient. La morbi-mortalité postopératoire était nulle. Le taux de conversion était nul. Après ligature portale, 5 sur les 6 patients avaient hypertrophié le FFR de façon significative, le gain moyen de volume du FFR était de 59,48% (31,02%-68,71%). Deux patients parmi les six avaient présenté une morbidité sévère après le deuxième temps hépatique (Clavien IIIb). La stratégie thérapeutique a été achevée chez tous les malades.

## Abstract (en)

Bilobar hepatic metastases from colorectal cancer pose a problem in terms of management, with curative surgery often requiring several stages. The purpose of our study was to evaluate laparoscopic approach with portal vein ligation in the first step of two-stage hepatectomy in the treatment of patients with synchronous liver metastases from colorectal cancers (SLMCRC). We conducted a single-center retrospective study from August 2016 to January 2020. It included patients with SLMCRC requiring two-stage curative surgery due to insufficient future liver remnant volume (FRL). The primary endpoint was to evaluate postoperative morbidity and mortality following first step laparoscopy at 30 days. The secondary endpoints were to evaluate conversion rate, FRL hypertrophy following laparoscopic portal vein ligation, postoperative morbidity and mortality of 2^nd^ step of two-stage hepatectomy and finally treatment completion rate. We included six patients (4 men and 2 women) with a mean age of 64 (44-72) years. The first step of surgery consisted of a laparoscopic colonic resection associated with right portal vein ligation in 5 patients and left portal vein ligation in one patient. The postoperative morbimortality was zero. The conversion rate was zero. After portal vein ligation, 5 of the 6 patients had significantly enlarged FRL, with a mean gain in FRL volume of 59.48% (31.02%-68.71%). Two of the six patients had severe morbidity after 2^nd^ step hepatectomy (Clavien IIIb). All patients completed the treatment.

## Introduction

Les métastases hépatiques bilobaires des cancers colorectaux posent un problème de prise en charge, la chirurgie curative nécessite souvent plusieurs temps [1] avec des artifices pour hypertrophier le FFR, notamment la ligature ou l´embolisation portale (EP) [2]. En situation synchrone, la chirurgie de la tumeur primitive doit être prise en compte dans la stratégie thérapeutique. L´approche laparoscopique pour la résection de la tumeur primitive et la ligature portale (LP) lors du premier temps pourrait être intéressante dans cette situation, la laparoscopie a un intérêt diagnostique puisqu´elle permet d´éliminer une carcinose péritonéale qui contre-indiquerait le geste hépatique, elle a aussi un intérêt thérapeutique puisqu´elle permet d´aborder les deux sites tumoraux en simultané de façon mini-invasive et réaliser ainsi une chirurgie de “downstaging” préparant la chirurgie hépatique majeure secondaire [3]. Notre étude avait pour but d´évaluer l´approche laparoscopique avec ligature portale dans le premier temps des hépatectomies en deux temps pour les métastases hépatiques synchrones des cancers colorectaux.

## Méthodes

**Conception de l´étude:** il s´agit d´une étude rétrospective monocentrique à partir de données recueillies de manière prospective qui s´est étalée d´aout 2016 à janvier 2020.

**Cadre de l´étude:** elle a été conduite au sein du service de chirurgie oncologique « A » de l´établissement hospitalier spécialisé Centre Pierre et Marie Curie d´Alger (Algérie). Il s´agit d´un centre tertiaire spécialisé en chirurgie oncologique notamment hépato-biliaire et colorectale.

**Critères d´inclusion:** nous avons inclus les patients qui présentaient un cancer colorectal avec des métastases hépatiques synchrones nécessitant une chirurgie curative en deux temps à cause d´une insuffisance du volume du FFR éligibles à une chirurgie par voie laparoscopique.

**Critères d´exclusion:** contre-indication relative ou absolue à l´abord laparoscopique.

**Les objectifs de l´étude:** l´objectif principal de notre étude était d´évaluer la morbi-mortalité postopératoire du premier temps laparoscopique à 30 jours . Les objectifs secondaires étaient d´évaluer le taux de conversion, l´hypertrophie du FFR après ligature portale laparoscopique, la morbi-mortalité postopératoire du 2^e^ temps hépatique et enfin d´évaluer le taux d´achèvement de la stratégie thérapeutique.

**Recueil des données:** les données démographiques des patients, ainsi que les données cliniques et paracliniques ont été consignées sur un fichier Excel, précisant notamment la localisation de la tumeur primitive et l´extension de la maladie métastatique hépatique selon les données de la tomodensitométrie et/ou l´imagerie par résonnance magnétique réalisant une cartographie précise des localisations secondaires hépatiques. C´est à partir de ces données que le dossier du patient était discuté en réunion de concertation pluridisciplinaire pour décider de la stratégie thérapeutique et chirurgicale. Les résultats obtenus concernant l´objectif principal et les objectifs secondaires de l´étude étaient répertoriés sur le même fichier Excel. L´exploitation et l´analyse informatique des données étaient faites grâce au logiciel IBM SPSS *statistics version 25*.

**Figure 1:**
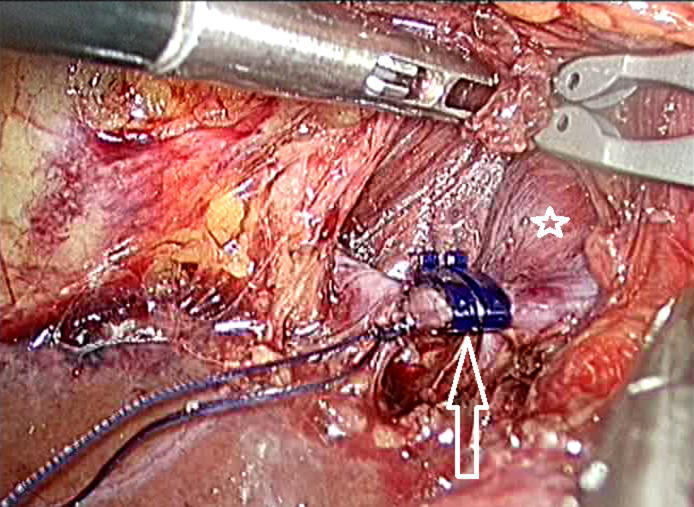
ligature portale droite par des clips verrouillés (étoile blanche sur la branche portale gauche, flèche blanche montrant deux clips verrouillés sur la branche portale droite)

**Protocole thérapeutique:** la résection de la tumeur colorectale primitive associée à une ligature portale étaient planifiées sous coelioscopie ce qui correspondait au premier temps chirurgical qui se faisait après une chimiothérapie d´induction (Il a été établi qu´un volume de FFR < 40% contre indiquerait la résection en un temps chez nos patients ayant reçu une chimiothérapie) [1]. La clearance du foie controlatéral à la ligature pouvait être associée et se faisait sous coelioscopie lors de ce premier temps. Quatre à cinq trocarts étaient utilisés lors du temps de résection colorectale, un trocart épigastrique était rajouté pour le temps hépatique. La ligature portale se faisait par l´application de deux clips verrouillés sur la branche portale concernée après identification du départ de la branche portale controlatérale (Figure 1). L´incision d´extraction des pièces opératoires était une incision de Pfannenstiel. Le deuxième temps chirurgical était planifié après une chimiothérapie d´intervalle et réévaluation morphologique qui appréciait le degré d´hypertrophie du FFR ainsi que l´absence de progression de la maladie métastatique.

Le volume du FFR, le volume total du foie, le volume du foie à réséquer ainsi que le volume tumoral ont été calculés avant et après ligature portale par scanner hélicoïdal triphasique, en utilisant un logiciel (Horos™ Viewer) de mesure de surface, délimitant dans chaque image numérisée le contour des segments hépatiques concernés par la mesure. La surface obtenue était multipliée par l'épaisseur de la coupe pour obtenir le volume de chaque section. Le calcul du taux d´hypertrophie du futur foie restant (HFFR) se faisait grâce à la formule suivante: % HFFR = (volume du FFR post ligature - volume du FFR de base) x100/ volume du FFR de base. L´HFFR était jugée adéquate si elle atteignait un niveau permettant une résection sure avec une intention curative, le ratio volume du FFR/poids du malade était calculé, un ratio > 0,5 [4] était considéré suffisant pour effectuer la résection, l´évaluation de l´hypertrophie se faisait par des scanners durant l´intervalle entre les deux interventions.

Le deuxième temps hépatique se faisait par laparotomie et consistait en une hépatectomie majeure qui permettait d´atteindre l´exérèse complète de la maladie métastatique hépatique. Les évènements indésirables périopératoires étaient notés, les incidents peropératoires étaient évalués par la classification de Oslo [5] qui avait modifié celle de Satava [6]. La morbidité était évaluée à 30 jours, les évènements pouvaient survenir au cours de l´hospitalisation ou après la sortie du patient. La gravité de la morbidité était évaluée selon la classification de Clavien-Dindo [7]. La mortalité était évaluée à 30 jours, la cause du décès dans cette éventualité était précisée. On avait considéré comme conversion, la réalisation par nécessité de toute la procédure chirurgicale (temps colorectal et temps hépatique) par laparotomie. L´achèvement de la stratégie thérapeutique menant à la résection curatrice était évalué pour chaque patient, en cas de « drop out » la cause d´abandon du protocole était précisée.

**Aspects éthiques et règlementaires:** la confidentialité et l'anonymat des patients opérés ont été respectés conformément aux principes de l'éthique et de la déontologie médicale. Les participants à l´étude ont été informés des différents aspects de la nouvelle approche laparoscopique et de la stratégie thérapeutique. Un consentement éclairé verbal a été obtenu avant l´inclusion.

## Résultats

Nous avons inclus six patients (4 hommes et 2 femmes), l´âge moyen était de 64 (44-72) ans, parmi eux 2 patients avaient des comorbidités à type de cardiopathie pour l´un (mise en place de stent coronarien) et de dilatations de bronches pour l´autre. Trois patients avaient un antécédent de chirurgie abdominale (2 par voie médiane, 1 par voie de Mac Burney). L´indice de masse corporelle (IMC) moyen était de 22(18-26) Kg/m^2^ Dans les Tableau 1 et Tableau 2 sont rapportés les types d´interventions réalisées, les volumétries avant et après ligature portale ainsi que les délais de réintervention. Au cours du premier temps, un incident peropératoire d´ordre anesthésique classé grade 1 d´Oslo a été noté chez le patient cardiopathe à type de troubles du rythme et d´extrasystoles à l´induction anesthésique traités médicalement. Aucune transfusion peropératoire n´a été notée chez les 6 patients. La morbi-mortalité postopératoire était nulle. Le taux de conversion était nul.

**Tableau 1:** hépatectomies en deux temps après ligature portale

	Tumeur primitive	Métastase hépatique (segments)	Première intervention (laparoscopie)	Deuxième intervention	Troisième intervention
**Patient 1**	Sigmoïde	VI,VII,VIII	RSB, LPD	Hépatectomie droite	_
**Patient 2**	Sigmoïde	II,III,V,VIII	RSB, Wedge résection III, LPD	Hépatectomie droite, métastasectomie II	_
**Patient 3**	Colon gauche	VI,VII,VIII	Colectomie gauche, LPD	Sectoriectomie postérieure élargie au VIII	_
**Patient 4**	Sigmoïde	III,V,VI,VII	RSB, métastasectomie III, LPD	Hépatectomie droite	_
**Patient 5**	Sigmoéde	II,IV,V,VIII	RSB, LPG	Hépatectomie gauche élargie au secteur antérieur droit (V, VIII)	_
**Patient 6**	Sigmoéde	II,III,VI,VII,VIII	RSB, Métastasectomie II, LPD	Partition hépatique	Hépatectomie droite

Ligature portale droite (LPD), Ligature portale gauche (LPG), Résection segmentaire basse (RSB)

**Tableau 2:** gain de volume après ligature portale et intervalle chirurgical

	Volume FFR initial	Volume FFR après ligature portale	Gain de volume	Délai entre les interventions	CT dans l’intervalle
**Patient 1**	394,58 cc	517 cc	31,02 %	28 semaines	Oui
**Patient 2**	383,34 cc	622 cc	62,25 %	12 semaines	Oui
**Patient 3**	312,2 cc	313 cc	0,26 %	20 semaines	Oui
**Patient 4**	382,84 cc	645,90 cc	68,71 %	16 semaines	Oui
**Patient 5**	304,52 cc	509 cc	67,14 %	13 semaines	Non
**Patient 6**	301,48 cc	373,35cc/ 507,38cc	23,84%/ 68,29%	1^ère^ -------2^è^ 08 semaines 2^è^------3^è^ 02 semaines	Oui


Futur foie restant (FFR), Chimiothérapie (CT)

Après ligature portale, 5 sur les 6 patients avaient hypertrophié le FFR de façon significative, le gain moyen de volume du FFR était de 59,48% (31,02%-68,71%). Une absence d´hypertrophie hépatique (gain de 0,26% de volume du FFR) a été notée chez le patient n°3, le deuxième temps hépatique avait été réalisé chez ce patient (sectoriectomie postérieure élargie au segment VIII) au lieu d´une hépatectomie droite, suite à une réduction importante du volume des métastases hépatiques sous traitement médicamenteux. Chez le patient n°6 un geste supplémentaire de partition hépatique était nécessaire après la ligature portale lors d´une deuxième intervention chirurgicale (à 8 semaines) (Tableau 2) à cause d´une hypertrophie insuffisante due au développement d´un cavernome porte avec revascularisation des territoires hépatiques exclus initialement et ce pour réaliser une hépatectomie majeure (hépatectomie droite) dans un troisième temps chirurgical (à 2 semaines) après une hypertrophie du FFR qui est passée de 23,84% à 68,29% entre la 2^e^ et la 3^e^ intervention. Pour le reste des malades la résection des métastases hépatiques avait été réalisée en deux temps.

**Figure 2:**
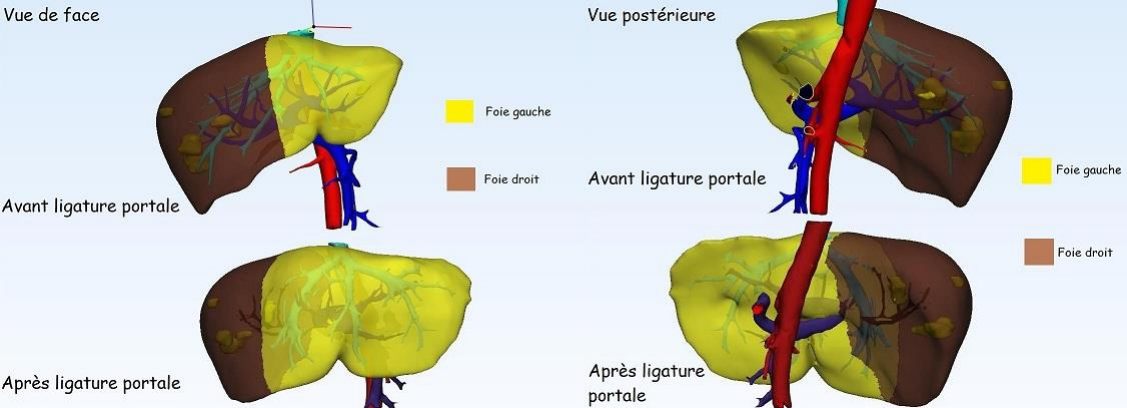
reconstruction 3D avec volumétrie montrant l´hypertrophie du foie gauche après ligature portale droite chez le patient n°4 (noter la cicatrice de métastasectomie (clearance du foie gauche))

La (Figure 2) montre sur une reconstruction 3D l´hypertrophie du FFR chez le patient n°4. Au total quatre hépatectomies droites avaient été réalisées dont une après une partition hépatique supplémentaire après la ligature portale, ainsi qu´une hépatectomie gauche élargie au secteur antérieur droit et une sectoriectomie postérieure élargie au segment VIII. Ces interventions avaient été réalisées par laparotomie (incision de Makuuchi), les adhérences liées à la première intervention n´étaient pas nombreuses, n´ayant pas entravé le déroulement de la seconde intervention. Deux patients parmi les six avaient présenté une morbidité sévère après le deuxième temps hépatique (Clavien IIIb) qui avait nécessité une reprise chirurgicale (collection profonde et péritonite biliaire). L´évolution postopératoire était favorable chez les deux patients. Un autre patient avait nécessité une transfusion postopératoire (Clavien II).

## Discussion

La réalisation du premier temps de résection par laparoscopie est faisable et sure dans la chirurgie en plusieurs temps des métastases hépatiques synchrones des cancers colorectaux, la morbi-mortalité dans notre courte série était nulle, en plus des avantages classiques de la chirurgie mini-invasive sur la réhabilitation celle-ci a permis un deuxième temps par laparotomie plus aisé en diminuant les adhérences postopératoires.

Nos patients avec des métastases hépatiques synchrones, étaient non résécables d´emblée et ne pouvaient être traités par une seule intervention chirurgicale. Une prise en charge onco-chirurgicale en plusieurs temps était nécessaire pour arriver à une résection complète de la néoplasie. Durant ce parcours, existait un risque réel de progression de la maladie métastatique, synonyme de la sortie du patient du projet thérapeutique ou “drop out” estimé dans les publications à environ un malade sur cinq. Dans sa série rétrospective monocentrique sur plus de 300 cas Yamashita [8] avait rapporté qu´approximativement 20% des patients ayant bénéficié d´une embolisation portale, n´étaient pas reséqués finalement, principalement pour progression de leur maladie. Cette sortie pourrait être aussi dans certains cas due à une hypertrophie insuffisante du foie malgré une embolisation ou ligature portale.

La comparaison de l´efficacité de la ligature portale par rapport à l´embolisation portale dans l´induction de l´hypertrophie hépatique a fait l´objet de plusieurs publications avec des résultats discordants, si certaines rapportent la supériorité de l´embolisation portale [9,10] expliquant sa plus grande efficacité par le fait qu´elle obstrue les vaisseaux contrairement à la ligature portale où les shunts peuvent revasculariser les territoires exclus initialement. D´autres publications ne retrouvent pas de différence entre les deux techniques [11], aussi les deux techniques peuvent être inefficaces par la formation d´un cavernome porte. La métanalyse publiée en 2017 par C.J. Isfordink [12], avait identifié 21 études éligibles pour l´analyse dans la littérature ayant inclus 1953 embolisations portales et 123 ligatures portales, il n´y avait pas de différence significative dans le taux d´hypertrophie du FFR entre les deux techniques (EP 43,2%, LP 38,5%, p = 0,39). Le nombre de résections hépatiques annulées pour une hypertrophie insuffisante était significativement moindre après ligature portale (p = 0,002). Il n´y avait pas de différence en post intervention de la mortalité ou de la morbidité.

Notre choix s´est porté sur la ligature portale pour plusieurs raisons: A) premièrement: parce qu´on était en situation synchrone, la tumeur primitive en place, ce qui impliquait un premier temps chirurgical colorectal. Ce temps chirurgical permettrait une exploration abdominale à la recherche d´une éventuelle contre-indication à la résection qui rendrait toute la stratégie sur le foie caduque. B) Deuxièmement: parce qu´on avait opté pour une chirurgie laparoscopique mini-invasive de ce premier temps qui permettrait la résection de la tumeur primitive et la ligature portale dans le même temps opératoire. C) Troisièmement: parce que l´embolisation portale est certes un geste mini-invasif mais non dénué de morbidité. Plusieurs complications ont été décrites dans la littérature [13], l´hémorragie, l´hématome, l´hémobilie, l´extension indésirable de l´embolisation vers des territoires qui ne devaient pas être embolisés (tronc porte par embolisation rétrograde, veine du segment IV), les thromboses veineuses, les complications infectieuses… *In fine* notre stratégie avait permis d´atteindre la résection complète des métastases hépatiques.

En analysant la littérature, les gains en volume du future foie restant (FFR) rapportés étaient variables, Robles [9] avait observé après ligature portale un gain médian de 30% (21%-60%), ces constatations étaient faites après un délai médian de 35 jours (28j-60j) , dans la métanalyse de Isfordink [12] le taux moyen d´hypertrophie après embolisation portale était de 43,2%, et de 38,5% après ligature portale, l´intervalle entre l´embolisation ou la ligature portale et la réévaluation de la volumétrie variait entres les études (2 à 8 semaines). D´autres résultats [14] concernant l´embolisation portale sont présentés dans le Tableau 3. Dans notre série 5 patients avaient hypertrophié le futur foie restant de façon significative, le gain moyen de volume du FFR était de 59,48%, un taux supérieur aux taux présentés par Isfordink. Ceci pourrait être expliqué par le délai plus long à l´intervention dans notre série qui était de 15,8 semaines en moyenne. Le comportement dans le temps du foie non embolisé a été étudié, il a été rapporté que le taux de régénération augmentait au cours des 3 premières semaines, suivait ensuite une phase en plateau avec une légère augmentation supplémentaire du volume [15]. La publication de Corrêa [16] s´était intéressée au devenir du foie sur une période d´une année après embolisation portale droite chez dix patients qui n´avaient pas été réséqués pour des raisons diverses.

**Tableau 3:** hypertrophie du foie gauche après embolisation portale droite [14]

Références	Foie non tumoral	Matériel	Délai	Hypertrophie
**Goto *et al*. 19**	Normal	Colle biologique	2 semaines	32 %
**Imamura *et al*. 20**	Normal et anormal	Colle biologique	2 semaines	31 %
**Tanaka *et al*. 21**	Fibrose F1 à F4	Colle biologique	2 semaines	37 %
**Harada *et al*. 22**	–	Colle biologique	3 semaines	44 %
**Elias *et al*. 23**	Normal	Colle acrylique	4 semaines	70 %
**Shimamura *et al*. 24**	Fibrose F1 à F4	Alcool	2 semaines 4 semaines	93 % 111 %

**Figure 3:**
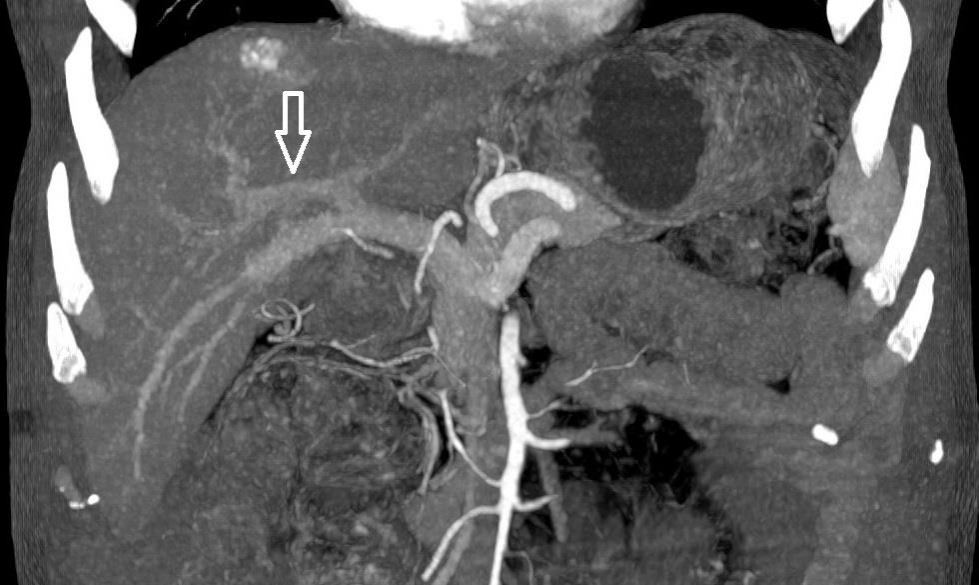
variation anatomique portale expliquant l´échec de la ligature portale chez le patient n°3 (flèche blanche montrant la veine sectorielle antérieure droite provenant de la branche portale gauche)

**Figure 4:**
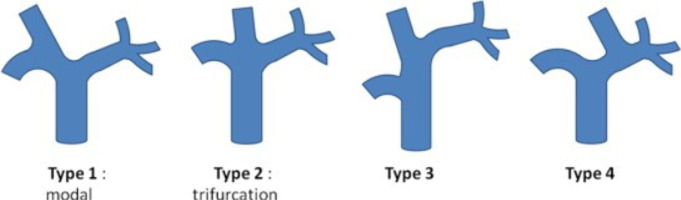
schémas des variations portales les plus fréquentes (selon Cheng)

Au cours de cette année, le foie gauche avait augmenté au total de 83,4%. Sur la croissance totale, 50% s´étaient produits 90 jours après l´embolisation et 75% après 230 jours. Ces données confirment que l'atrophie du côté traité et l'hypertrophie compensatrice du côté controlatéral se produisent continuellement jusqu'à au moins 360 jours après l´embolisation. Donc dans notre série l´allongement du délai à l´intervention avait eu un effet positif sur le taux d´hypertrophie mais en parallèle le risque d´une progression de la maladie existait. L´absence d´une hypertrophie du FFR avait été constatée chez le patient n°3 (Figure 3), une cause purement anatomique avait été identifiée, en fait une variation anatomique de la distribution portale type 4 de Cheng (Figure 4) existait chez ce patient. Pour rappel dans cette variation type 4 de Cheng la veine sectorielle antérieure droite provient de la branche portale gauche et se voit dans (0,9-6,4%) de la population [17]. La branche portale qui avait été clippée chez ce patient n´irriguait que le secteur postérieur droit. La privation veineuse hépatique (PVH) a été proposée comme alternative, technique qui combine à la fois l´embolisation portale et sus-hépatique pour augmenter et accélérer l´hypertrophie hépatique. Guiu *et al*. ont rapporté une série de sept patients avec un succès technique et une hypertrophie induite chez tous les patients [18]. Cependant, d´autres études sont nécessaires afin d´évaluer l´importance clinique de la PVH par rapport à l´EP [18].

## Conclusion

L´approche laparoscopique est faisable lors du premier temps des hépatectomies en deux temps pour les métastases hépatiques synchrones des cancers colorectaux, elle représente l´étape mini-invasive du traitement curateur préparant ainsi une chirurgie hépatique majeure.

### Etat des connaissances sur le sujet

- *L´hépatectomie en deux temps est indiquée dans certains cas de métastases bilobaires;*
- *La ligature portale ou l´embolisation portale assurent souvent une hypertrophie du FFR;*
- *La résection R0 améliore la survie*.

### Contribution de notre étude à la connaissance

- *La laparoscopie est faisable lors du premier temps des hépatectomies en deux temps lorsque la tumeur primitive est en place car elle permet d´aborder les deux sites tumoraux avec un minimum de traumatisme pariétal et sans majorer la morbidité;*
- *La ligature portale par laparoscopie est efficace sur l´hypertrophie du FFR et peut être une alternative à l´embolisation portale en situation synchrone;*
- *L´approche laparoscopique dans le premier temps facilite l´hépatectomie majeure du deuxième temps*.

## References

[1] Adam R, Laurent A, Azoulay D, Castaing D, Bismuth H (2000). Two-stage hepatectomy: a planned strategy to treat irresectable liver tumors. Annals of Surgery.

[2] Makuuchi M, Thai BL, Takayasu K, Takayama T, Kosuge T, Gunvén P (1990). Preoperative portal embolization to increase safety of major hepatectomy for hilar bile duct carcinoma: a preliminary report. Surgery.

[3] Pessaux P, Panaro F (2009). Advantages of the first-step totally laparoscopic approach in 2-staged hepatectomy for colorectal synchronous liver metastasis. Surgery.

[4] Truant S, Oberlin O, Sergent G, Lebuffe G, Gambiez L, Ernst O (2007). Remnant Liver Volume to Body Weight Ratio ≥0.5%: a new cut-off to estimate postoperative risks after extended resection in noncirrhotic liver. Journal of the American College of Surgeons.

[5] Kazaryan AM, Røsok BI, Edwin B (2013). Morbidity Assessment in Surgery: refinement proposal based on a concept of perioperative adverse events. ISRN Surgery.

[6] Satava RM (2005). Identification and reduction of surgical error using simulation. Minimally Invasive Therapy & Allied Technologies.

[7] Clavien PA, Barkun J, de Oliveira ML, Vauthey JN, Dindo D, Schulick RD (2009). The clavien-dindo classification of surgical complications: five-year experience. Annals of Surgery.

[8] Yamashita S, Sakamoto Y, Yamamoto S, Takemura N, Omichi K, Shinkawa H (2017). Efficacy of preoperative portal vein embolization among patients with hepatocellular carcinoma, biliary tract cancer, and colorectal liver metastases: a comparative study based on single-center experience of 319 cases. Ann Surg Oncol.

[9] Robles R, Marín C, Lopez-Conesa A, Capel A, Perez-Flores D, Parrilla P (2012). Comparative study of right portal vein ligation versus embolisation for induction of hypertrophy in two-stage hepatectomy for multiple bilateral colorectal liver metastases. European Journal of Surgical Oncology (EJSO).

[10] van den Esschert JW, van Lienden KP, de Graaf W, Maas MAW, Roelofs JJTH, Heger M (2011). Portal vein embolization induces more liver regeneration than portal vein ligation in a standardized rabbit model. Surgery.

[11] Capussotti L (2008). Portal vein ligation as an efficient method of increasing the future liver remnant volume in the surgical treatment of colorectal metastases. Arch Surg.

[12] Isfordink CJ, Samim M, Braat MNGJA, Almalki AM, Hagendoorn J, Borel Rinkes IHM (2017). Portal vein ligation versus portal vein embolization for induction of hypertrophy of the future liver remnant: a systematic review and meta-analysis. Surgical Oncology.

[13] van Gulik TM, van den Esschert JW, de Graaf W, van Lienden KP, Busch ORC, Heger M (2008). Controversies in the use of portal vein embolization. Dig Surg.

[14] Farges O, Denys A (2001). Embolisation portale avant hépatectomie: techniques, indications et résultats. Annales de Chirurgie.

[15] Nagino M, Ando M, Kamiya J, Uesaka K, Sano T, Nimura Y (2001). Liver regeneration after major hepatectomy for biliary cancer: liver regeneration after major hepatectomy. Br J Surg.

[16] Corrêa D (2010). Kinetics of Liver Volume Changes in the First Year After Portal Vein Embolization. Arch Surg.

[17] Cheng YF, Huang TL, Lee TY, Chen TY, Chen CL (1996). Variation of the intrahepatic portal vein; angiographic demonstration and application in living-related hepatic transplantation. Transplant Proc.

[18] Guiu B, Chevallier P, Denys A, Delhom E, Pierredon-Foulongne M-A, Rouanet P (2016). Simultaneous trans-hepatic portal and hepatic vein embolization before major hepatectomy: the liver venous deprivation technique. Eur Radiol.

[19] Goto Y, Nagino M, Nimura Y (1998). Doppler estimation of portal blood flow after percutaneous transhepatic portal vein embolization. Annals of Surgery.

[20] Imamura H, Shimada R, Kubota M, Matsuyama Y, Nakayama A, Miyagawa S (1999). Preoperative portal vein embolization: an audit of 84 patients. Hepatology.

[21] Tanaka H, Hirohashi K, Kubo S, Ikebe T, Tsukamoto T, Hamba H (1999). Influence of histological inflammatory activity on regenerative capacity of liver after percutaneous transhepatic portal vein embolization. Journal of Gastroenterology.

[22] Harada H (1997). Fate of the human liver after hemihepatic portal vein embolization: cell kinetic and morphometric study. Hepatology.

[23] Elias D, Cavalcanti A, de Baere T, Roche A, Lasser P (1999). Long-term oncological results of hepatectomy performed after selective portal embolization. Ann Chir.

[24] Shimamura T, Nakajima Y, Une Y, Namieno T, Ogasawara K, Yamashita K (1997). Efficacy and safety of preoperative percutaneous transhepatic portal embolization with absolute ethanol: a clinical study. Surgery.

